# Environmental Drivers of an Intertidal Bryozoan Community in the Barents Sea: A Case Study

**DOI:** 10.3390/ani12050552

**Published:** 2022-02-23

**Authors:** Olga Yu. Evseeva, Tatyana G. Ishkulova, Alexander G. Dvoretsky

**Affiliations:** Murmansk Marine Biological Institute, Russian Academy of Sciences (MMBI RAS), 183010 Murmansk, Russia; evseeva@mmbi.info (O.Y.E.); ishkulova@mmbi.info (T.G.I.)

**Keywords:** bryozoa, biodiversity, biomass, intertidal zone, driving factors, Barents Sea

## Abstract

**Simple Summary:**

Bryozoans are colonial organisms that are usually found attached to solid substrata. They are known to be common components of benthic communities from the littoral zone to deep-sea areas. Despite the long history of bryozoan research in the Barents Sea, intertidal communities of this group are less studied than those at open water sites. This is particularly true for the assessment of the role of environmental factors in diversity and biomass fluctuations of Arctic bryozoan assemblages. We collected bryozoans at two adjoining coastal sites in the southern Barents Sea and detected range extension of one boreal species, which reflects a warming trend and borealization of the benthic fauna in the region. In general, the total bryozoan biomass and diversity were lower than in deep-water sites of the sea. Salinity and temperature were found to be the main predictors of bryozoan species richness and biomass, respectively, with more diverse and abundant assemblages occupying habitats with higher salinity and lower temperature. Our findings are in accordance with a general opinion that benthic communities of the coastal Barents Sea are mainly controlled by temperature regime fluctuations and freshwater runoffs.

**Abstract:**

There is a lack of knowledge regarding the modern status of intertidal bryozoan communities in the coastal Barents Sea. Here, we studied species composition, richness, and biomass of bryozoans in Yarnyshnaya and Dalnezelenetskaya Bays, both located in the eastern part of the Kola Peninsula (Barents Sea), in summer. Species composition and biodiversity were consistent with previous research but the record of the ctenostome bryozoan *Walkeria* *uva* is the first for the region indicating eastward range expansion of this species associated with climate forcing in the Arctic. Mean biomass was relatively low accounting for 2.25 ± 0.95 g·m^−2^. The most common species were *Eucratea loricata*, *Harmeria scutulata*, *Crisia eburnea*, and *Cribrilina cryptooecium* averaging 96% of the total biomass. Cluster analysis delineated two distinct groups of stations, one with true marine conditions and another with brackish water conditions. Redundancy analysis revealed that bryozoan diversity was strongly associated with salinity fluctuations being extremely low at brackish water sites. In contrast, water temperature was found to be a significant contributor to biomass with the lowest values found at warmer waters probably owing to the predominance of Boreo-Arctic species which prefer lower-temperatures. Other hydrological variables (dissolved organic matter, silicates, and oxygen) were consistent with usual summer values and had no significant effects on the bryozoan assemblages. Our study provides a reference point for further biodiversity studies in changing marine ecosystems of the Arctic region.

## 1. Introduction

Interactions of the cold Arctic and warm Atlantic waters is the main feature of the Barents Sea [1] making this large marine ecosystem the most productive shelf region of the Arctic [2,3] and supporting abundant stocks of fish and shellfish [4,5,6]. The Barents Sea, like other Arctic regions, is being influenced by the effects of global warming and we are now witnessing significant shifts in the ecosystem components and processes including strong salinification of the upper Eurasian Basin, attendant reductions in stratification, and altered nutrient fluxes and primary production [7,8,9].

The Arctic benthic fauna consists of a relatively small number of species (4800), many of which are eurythermal and may have successfully reached the North Polar Regions from the Pacific or boreal Atlantic [10]. Arctic food webs are believed to have poor annual primary production with clear seasonality. The zoobenthos are largely decoupled from the pelagic zone and most Arctic organisms grow very slowly due to harsh environmental conditions, such as low-temperatures and a decreased availability of food sources [11].

The lophotrochozoan phylum Bryozoa is a group of predominantly colonial, filter-feeders of approximately 6000 living species [12], inhabiting both marine and freshwater environments, and distributed from polar regions to tropics and from intertidal to abyssal depths [13,14,15,16,17]. The individual units of a bryozoan colony, also called “zooids”, are generally about 0.5–1 mm in size. Each zooid comprises soft parts (polypide) and a calcified or less often non-calcified body wall (cystid). Bryozoan colonies or zoariums are diverse in form, ranging from flat encrusting habits, where all zooids adhere to a hard substrate, to erect, rigid colonies with foliaceous, arborescent, or fenestrate shapes. Others build erect, flexible colonies, having chitinous joints between stem segments and chitinous rootlets for attachment. Still, others are free-living with bun- or disc-shaped forms [18].

In the Arctic, like in other regions, Bryozoa very often dominate communities in rocky and boulder habitats and on epiphytes [19,20,21]. They also colonize living hard substrata, such as gastropod shells occupied by hermit crabs [22] and carapaces of lithodid crabs [23,24,25] and spider crabs [26,27], as well as macrophytes [28].

Bryozoa are considered to be one of the most species-rich and diverse groups of the Arctic benthos [19,29,30]. The lowest species richness (98 species) is documented in the Canadian Arctic while the highest richness (328 species) is registered in the Barents Sea [21]. In Arctic seas, this phylum was established to be the fourth group by the number of species followed by polychaetes, crustaceans, and mollusks, except for the Chukchi Sea, where they have the highest species richness among all other major benthic taxa [21].

In recent decades, many studies focused on evaluating environmental drivers of bryozoan diversity in several regions such as the Barents, Chukchi, Kara, and East Siberian Seas [20,21,31,32,33,34]. The availability of suitable substrates, sedimentary perturbation, depth of sampling, latitude, and water-temperature were tested as potential drivers of bryozoan diversity in Arctic regions and the contribution of these factors was found to differ regionally and seasonally [20,34]. However, there are only a few studies focused on the role of hydrological conditions in driving bryozoan assemblages in the Arctic shoreline [11,20].

The aim of our research was to assess the contribution of different environmental factors to the composition of bryozoan communities in the intertidal zone in the eastern part of the Kola Peninsula (Barents Sea).

## 2. Materials and Methods

### 2.1. Study Area

**Figure 1:**
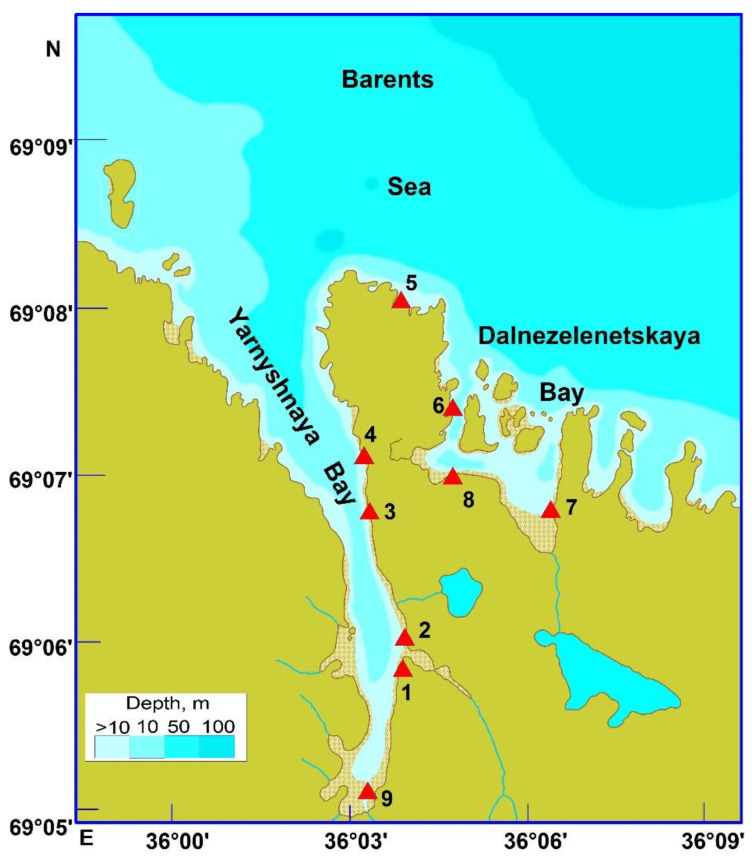
Location of sampling stations (triangles) and their numbers in the coastal Barents Sea in August 2014.

We studied bryozoans that occurred intertidally, on rocks, boulders, and macrophytes at 9 stations located in Yarnyshnaya Bay and Dalnezelenetskaya Bay (eastern part of the Kola Peninsula) in August 2014 (Figure 1).

Yarnyshnaya Bay is an open, relatively large gulf (6 km length and 2 km width) elongated from the north to the south. Rocky shores in the bay entrance are steep (the inclination of the coastline is 50–70°) while in the middle part, they alternate with boulders and become more gentle (25–35°), and at the head of the bay, grounds are composed of mud and sand with gravel and boulders with a 5–10° inclination of the coastline. The northerly direction is the prevailing wind direction in the bay. The intertidal zone at the head of Yarnyshnaya Bay is characterized by intense mixing of seawater. Storms prevail in the autumn–winter period. Tidal levels are higher than in Dalnezelenetskaya Bay (4.5 m). There is a strong increasing salinity gradient along the south–north direction (28–34 psu). The highest temperature level in the surface layer occurs in August (+8 °C) and the minimum in February (−1 °C). Ice coverage is registered in cold years only [35].

Dalnezelenetskaya Bay is a semi-closed relatively small gulf with five islands separating the area from the open sea. This site is almost square (2 × 2 km) with a total area of 2.23 km^2^ [36]. The maximum depths are registered in the western part of the bay. Mean depth is about 7 m. Tidal levels are high enough (3–4 m) to ensure intensive water exchange between the inner part of the bay and the open sea. The lowest temperature value in the surface layer (0.7 °C) occurs in February and the highest (9.7 °C)—in August. Salinity minimum (32.2 psu) is associated with high input of meltwater and usually is registered in May. In autumn and winter, salinity is quite stable averaging 34 psu [36]. The minimum level of dissolved oxygen is registered in December, the maximum in May [37].

Our stations covered areas with different wave regimes. The latter were classified according to Guryanova et al. [38] as follows: 1—the highest wave flow intensity, typical for oceanic coasts; 2—high constant wave flow intensity, typical for open coasts of gulfs; 3—medium wave flow intensity, typical for semi-closed coasts of gulfs; 4—weak wave flow intensity, typical for protected coasts of bays; 5—very low or zero wave flow intensity, typical for closed bays. Wave flow intensity indices (WFII) for our sampling stations were obtained from published sources [39,40].

### 2.2. Sampling and Processing

At each site of the study area, water samples were collected at low tide, in triplicate in glass containers rinsed with distilled water and oven dried. Concentrations of nitrates, phosphates, and silicates were measured using a PE-5300VI spectrophotometer. Inorganic dissolved phosphorus (P-PO_4_) was determined by the Murphy–Riley method [41], dissolved silicates (Si-SiO_3_) by the Korolev method [42], nitrogen (N-NO_2_ and N-NO_3_) by the Bendschneider and Robinson method [43], and oxygen by the Winkler method [44]. Seawater temperature and salinity were determined using a portable profiler.

At each station, bryozoans were collected at the same substrata (rock, boulders, and macrophytes) in triplicate from a 50 × 50 cm quadrate. They were counted and dissected off their substrata. The material was fixed in 4% buffered formalin. Small boulders covered with bryozoan colonies were transferred to the laboratory for further analysis. In the laboratory, bryozoans were identified under an MBS-10 stereomicroscope (OAO LZOS, Lytkarino, Russia) using the monographs of Kluge [29,30] and more recent publications when necessary. Bryozoan biomass was determined and expressed in g·m^−2^. Diversity of the bryozoan community was estimated using the Shannon index [45] calculated from the species biomass: H′ = −Σp_i_·log_2_p_i_ where i is the sample number and p_i_ is the proportion of the total biomass represented by the ith species. Pielou’s evenness index was calculated as J’ = H’/log_2_**S** [46], where S is the total number of species in a sample.

### 2.3. Statistical Analysis

Prior to statistical analyses, the data were checked for normality and homogeneity using the Shapiro–Wilks test and modified Levene’s test, respectively. The data were square-root-transformed when required.

Bryozoan community analysis was performed using multivariate statistics in the software package PRIMER 5.0 (PRIMER-E Ltd., Plymouth, UK). Biomass was square-root-transformed to decrease the weight of dominant species. Cluster analysis was used to distinguish the spatial communities based on the Bray–Curtis similarity measure and group average linkage classification. Similarities between station groups based on hierarchical clustering were tested using analysis of similarities (ANOSIM) in which global R = 1 indicates complete separation of groups and global R = 0 indicates no separation [47]. Species responsible for differences between the station groups were identified with SIMPER analysis [47].

Relationships between local environmental variables and diversity and biomass of bryozoans were examined using a Redundancy Analysis (RDA). Detrended correspondence analysis was used a priori to reveal whether the data ordination method was linear (appropriate for RDA analysis) or unimodal (appropriate for canonical correspondence analysis). The length of the first axis was <3 standard deviation units, showing the linear ordination method to be preferable [48]. The input dataset of environmental variables included water temperature, salinity, concentrations of nitrites, nitrates, phosphates, dissolved silicon, dissolved oxygen, as well as WFII. Two datasets were used for response variables: the first included biomasses of all species and the total biomass while the second included species richness (total and calculated for different construction forms and orientation of colonies), H’, and J’. A Monte Carlo permutation test (*n* = 999) was used to reveal the explanatory variables that best explained the bryozoan biomass and diversity data. All ordinations were performed using CANOCO for Windows v. 4.5 (Microcomputer Power, Ithaca, NY, USA) [48]. Mean values are presented with standard errors.

## 3. Results

### 3.1. Environmental Conditions

**Figure 2:**
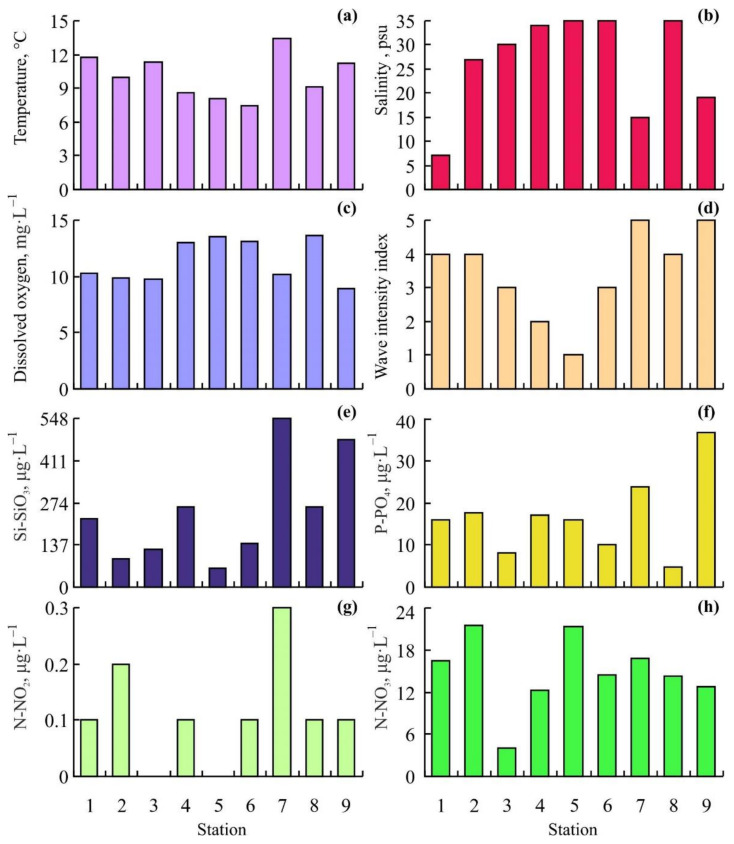
Variations in environmental conditions in the study area. (**a**)—temperature, (**b**)—salinity, (**c**)—oxygen concentration, (**d**)—wave flow intensity index, (**e**)—silicate concentration, (**f**)—phosphate concentration, (**g**)—nitrite concentration, (**h**)—nitrate concentration.

Environmental characteristics of the study area are presented in Figure 2.

The lowest water temperatures were detected at Stations 6 (7.4 °C) and 5 (8.1 °C) located in the entrance of Dalnezelentskaya Bay while the highest value was registered at Station 7 (13.4 °C) located at the beach of the bay. Decreased salinity levels (7, 15, 19, and 27 psu) were found at Stations 1, 7, 9, and 2, respectively. The highest wave flow intensity was registered at open Stations 4 and 5 while zero wave intensity occurred at the head of Dalnezelentskaya and Yarnyshnaya Bays (Stations 7 and 9). Oxygen concentrations accounted for 8.9–10.3 mg·L^−1^ at stations 1, 2, 3, 7, and 9 and 13.0–13.6 mg·L^−1^ at the rest stations. The highest phosphate and silicate concentrations were obtained from Stations 7 (23.9 and 547.6 μg·L^−1^, respectively) and 9 (36.6 and 477.7 μg·L^−1^, respectively). Nitrite concentrations were low ranging from 0 μg·L^−1^ (Stations 3 and 5) to 0.3 μg·L^−1^ (Station 7). The minimum level of nitrates (4 μg·L^−1^) was found at Station 3 whereas the maximum levels (21.3–21.5 μg·L^−1^) were registered at Stations 2 and 5.

### 3.2. Bryozoan Diversity, Biomass, and Community

A total of 25 species of bryozoans belonging to 2 classes, 3 orders, 19 families, and 22 genera were identified (Table 1).

**Figure 3:**
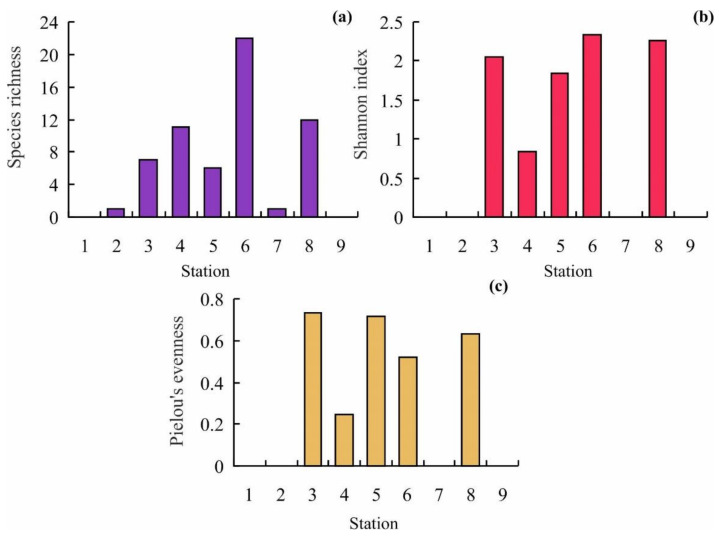
Variations in bryozoan diversity in the study area. (**a**)—species richness, (**b**)—Shannon index, (**c**)—Pielou’s evenness.

The most diverse order was Cheilostomatida (16 species, 64%). Biogeographic affinity of bryozoans registered in the study area indicates the predominance of Boreo-Arctic species (68%). The proportions of boreal and Arctic species were 24 and 8%, respectively. With regard to construction forms, flexible bryozoans (4 species, 16%) were found less frequently than calcified ones (21 species, 84%) and with respect to orientation of colonies, the proportion of encrusting bryozoans (17 species, 68%) was twice as much as that of erect ones (8 species, 32%). No bryozoan colonies were found at Stations 1 and 9. Six species, *Callopora weslawski* Kuklinski and Taylor, 2006, *Celleporella hyaline* (Linnaeus, 1767), *Cribrilina cryptooecium Norman, 1903*, *Electra pilosa* (Linnaeus, 1767), *Flustrellidra hispida* (O. Fabricius, 1780), and *Harmeria scutulata* (Busk, 1855) were the most frequent occurring at >50% of the rest sampling stations. Maximum species richness (SR) was recorded at Station 6 (22 species) while the minimum was at Stations 2 and 7 (1 species); Pielou’s evenness index (J’) ranged from 0.24 at station 4 to 0.71 at Station 5; Shannon’s diversity index H’ varied from 0 at Stations 2 and 7 to 2.33 at Station 6 (Figure 3). Mean values for SR, J’, and H’ indices were 6.7 ± 2.5, 0.56 ± 0.09, and 1.33 ± 0.39, respectively.

Mean biomass values and frequency of all species were low (Table 1). Overall, 96% of the total biomass was provided by 9 species, among which relatively high values in the whole material were noted only for *Eucratea loricata* (Linnaeus, 1758), *Harmeria scutulata*, *Crisia eburnean* (Linnaeus, 1758), and *Cribrilina cryptooecium* (Table 1). The averaged bryozoan biomass in the study area was calculated to be 2.25 ± 0.95 g·m^−2^, with the highest values at Stations 4 (5.49 g·m^−2^) and 6 (7.92 g·m^−2^).

**Figure 4:**
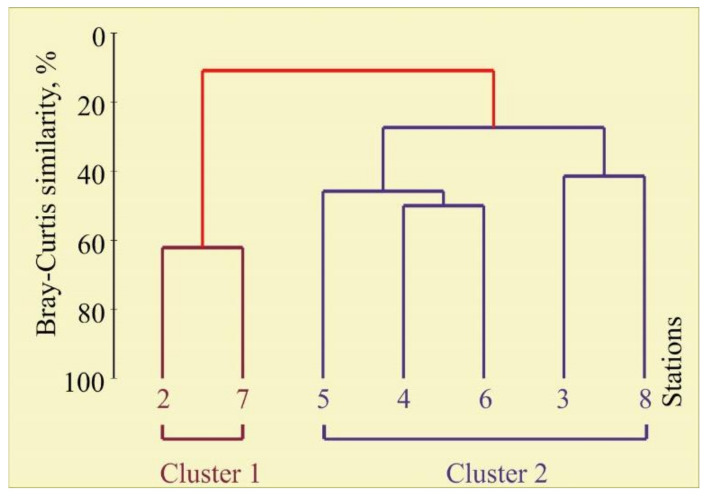
Dendrogram resulting from clustering performed on the Bray–Curtis similarity matrix produced from the square-root transformed bryozoan biomass data in the coastal Barents Sea in August 2014.

The cluster analysis of the bryozoan community composition and biomass revealed two groups at the 11% similarity level (Figure 4), clearly separating Stations 2 and 7 (Cluster 1) from all other stations (Cluster 2).

Cluster 1 included the stations where only one species (*Flustrellidra hispida*) was detected. Cluster 2 represented stations with high biomass and diversity. The ANOSIM test used to compare the composition between the two clusters indicated that there was a significant dissimilarity of bryozoan biomass (global R = 0.80, *p* = 0.048). A SIMPER analysis distinguished species that most contributed to the separation of each cluster grouping. Taxa contributing most to the dissimilarity between Cluster 1 and 2 included 9 species (89% cumulative dissimilarity) (Table 2).

### 3.3. Relationships between Bryozoan Data and Environmental Variables

**Figure 5:**
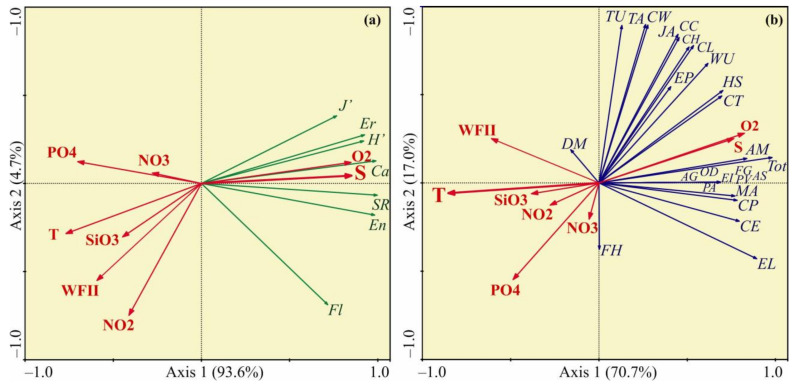
Ordination of samples by redundancy analysis with respect to bryozoan diversity (**a**) and biomass (**b**) and their relations to environmental variables in the coastal Barents Sea in August 2014. The proportions of the total variability explained by the first two axes are given. Biological variables: AG—*Amathia gracilis*, AM—*Alcyonidioides mytili*, AS—*Aquiloniella scabra*, CC—*Cribrilina cryptooecium*, CE—*Crisia eburnea*, CH—*Celleporella hyalina*, CL—*Callopora lineata*, CP—*Crisiella producta*, CT—*Cylindroporella tubulosa*, CW—*Callopora weslawski*, DM—*Dendrobeania murrayana*, EI—*Escharella immersa*, EL—*Eucratea loricata*, EP—*Electra pilosa*, FG—*Filicrisia geniculata*, FH—*Flustrellidra hispida*, HS—*Harmeria scutulata*, JA—*Juxtacribrilina annulata*, MA—*Microporella arctica*, OD—*Oncousoecia diastoporides*, PA—*Porella alba*, PV—*Patinella verrucaria*, TA—*Tegella arctica*, TU—*Tegella unicornis*, WU—*Walkeria uva*, Tot—total biomass, SR—total species richness, Ca—species richness of calcified bryozoans, Fl—species richness of flexible bryozoans, En—species richness of encrusting bryozoans, Er—species richness of erect bryozoans, H’—Shannon index, J’—Pielou’s evenness. Environmental variables: T—temperature (°C), S—salinity, O_2_—oxygen concentration (mg L^−1^), SiO_3_—silicates (µg·L^−1^), PO_4_—phosphates (µg·L^−1^), NO_2_—nitrites (µg·L^−1^), NO_3_—nitrates (µg·L^−1^), and WFII—wave flow intensity index.

The RDA based on diversity indices of the bryozoan fauna showed that the first two axes explained more than 97% of the total variation in biomass. The diversity indices demonstrated a positive association with Axis 1 (Figure 5a) suggesting an increase in these parameters at colder-temperatures and higher-salinities. Forward selection procedure indicated that water salinity was the main factor that significantly contributed to the RDA model (Table 3).

The RDA based on bryozoan biomass indicated that only the first axis explained a large proportion of the variance in the data (70.7%). The ordination biplot showed the first axis was closely negatively correlated with water temperature and positively with salinity and oxygen concentration (Figure 5b). Most of bryozoan species were positively related with Axis 1, indicating that their biomasses tended to increase at the sites with higher-salinity and lower-temperature. Forward selection procedure found that water temperature was the only factor that significantly contributed to the RDA model (Table 3).

## 4. Discussion

### 4.1. Environmental Conditions

In the coastal Barents Sea, the summer hydrological regime strongly depends on tidal dynamics, inflows of warmer waters, and freshwater runoffs [1]. In August, a second phytoplankton bloom is usually registered in the Barents Sea resulting in an increased oxygen supply [49]. In coastal ecosystems, the biogeochemical role of phytoplankton primary production is to transform and incorporate reactive inorganic elements into organic forms, and these transformations are rapid and lead to measurable geochemical change during blooms. Examples include the depletion of inorganic nutrients (N, P, Si), supersaturation of oxygen, and removal of carbon dioxide [50,51]. This is the reason why the oxygen concentration was high at all our stations in the study period. The sites facing the open sea (Stations 5, 6, and 7) and, therefore, affected by high tidal and wave dynamics demonstrated the lowest temperature and the highest salinity levels in contrast to sites where wave dynamics were lower (Stations 1, 7, and 9) providing a clear stratification of water masses with the warmer upper layer and the colder lower layer. In addition, the latter stations were affected by freshwater runoffs from local creeks. August is the period when river runoffs are greatest resulting in high concentrations of dissolved biogenic elements [52]. Indeed, a strong discharge of fresh waters in the mentioned sites led to higher concentrations of dissolved biogenic elements in comparison to other sites. In general, the concentrations of phosphates, silicates, nitrites, and nitrates did not exceed the ranges established for these compounds by previous studies [52,53,54] indicating usual habitat conditions for bryozoans in the study period.

### 4.2. Bryozoan Diversity, Biomass, and Community

Previous research revealed the presence of 125 bryozoan species in the study area and adjacent waters including the subtidal zone [55,56]. Thus, our species richness accounted for 15% of the total diversity. The low species richness detected for the intertidal-zone bryozoan community is not surprising because, in the Barents Sea, the highest number of bryozoan species is registered at depths between 50 and 100 m due to the predominance of suitable substrata in this depth range [19]. Two species were the first records in this region: *Callopora* *weslawski*, an encrusting bryozoan species with a calcified body, and *Walkeria* *uva*, a flexible erect species. The former species was described in 2006 by Kuklinski and Taylor [57] and its absence in coastal waters of the Kola Peninsula may be attributed to misidentification as *Callopora whiteavesi*, which is suggested to have an Arctic circumpolar distribution [29,30]. The latter species is new to the bryozoan fauna of the eastern part of the Kola Peninsula but it occurs at sites located to the west of Kola Bay (Kluge [29]). As this species has a boreal origin, we can hypothesize that its finding is associated with recent warming in the Arctic. Indeed, a pronounced increase in water temperatures (both summer and averaged ones) was registered in the coastal Barents Sea in the period from 2001 to 2013 reflecting a general pattern of climate forcing in the sea [58]. Range expansion and invasions of boreal species is an expected consequence of rapid and unforeseen changes in the Arctic climate system which are mediated by increased inflows from the northern North Atlantic [59] and the new distribution record of *Walkeria* *uva* is in line with ecosystem changes called “borealization” of the Arctic, i.e., an increase in the relative importance of boreal organisms in local ecosystems due to poleward expansion of boreal species and a decrease in abundance of Arctic species [8].

In general, bryozoan biomass in the study area (2.3 g·m^−2^ ) was lower than reported in Is-Fjord (West Spitsbergen), where the maximum biomass varied from 3.1–4.2 at depth 160–230 m [60], in the central part of the sea where mean biomass amounted to 13 g·m^−2^ [61], and in Tykhaya Bay of the Guker Island (Franz-Josef Land), where a maximum of biomass reached 485 g·m^−2^ [62], but higher than in the northeastern part of the Barents Sea where the total biomass did not exceed 1 g·m^−2^ at most stations [63]. It is obvious that such variations reflect different environmental conditions within the contrasting ecological system of the sea.

The bryozoan community of brackish water habitats (Stations 2 and 7) was composed of only one species, *Flustrellidra hispida*. This ctenostome bryozoan has an amphiboreal distribution in the northern Atlantic from the White Sea and Barents Sea to the northwestern coast of France and from the St. Lawrence Gulf to Woods Hole, and in the northern Pacific from the Kuril Islands to Aleutian Islands, and from the Gulf of Alaska down to California [29,30,64]. This species can tolerate major salinity fluctuations, which is confirmed by their presence in areas strongly affected by freshwater discharges, such as the White Sea [64] and shallow-water sites of Gren-fjord, Svalbard [65]. The community found at the remaining stations with true marine environmental conditions (high-salinity and low-temperature) demonstrated the structure close to other Barents Sea sites [60,61,62,63] adjusted for the specific features of the littoral zone.

### 4.3. Relationships between Bryozoan Data and Environmental Variables

Kuklinski et al. [20] suggested that Svalbard bryozoan assemblages are driven by processes related to depth and sediment characteristics with similar species richness in shallow- and deep-water habitats but different dominant taxa. We sampled bryozoans on the same substrata and within the littoral zone, demonstrating that substrate availability and depth were irrelevant factors in our case.

As mentioned above, dissolved organic matter levels were in good accordance with normal values registered in the study area. This is the reason why these factors resulted in no clear relationships with bryozoan diversity and biomass. It is accepted that wave regimes could affect distribution and biodiversity patterns of bryozoans because many species tend to avoid habitats with high water dynamics [13,66]. Our data, however, do not support this assumption. More likely, this result is associated with the predominance of typical encrusting species with calcified bodies which can easily tolerate significant fluctuations in seawater turbulence [67]. In our study, many species were found at stations with high wave flow intensity in rock crevices. Ryland [13] suggested that some flexible taxa, such as *Alcyonidium* and *Flustrellidra* are known to occur more frequently in the intertidal zone where they attach themselves to macroalgae. In our study, only one species with encrusting branches, *Dendrobeania* *murrayana*, was found attached to *Saccharina latissima* thalli at Station 3 with moderate wave flow intensity. Additionally, intense water currents are crucial for filter-feeding organisms, such as bryozoans [68].

Denisenko and Grebmeier [34] revised species richness of bryozoans in the Chukchi Sea and concluded that temperature gradients across geographical zones control fauna richness. We made the same conclusion when discussing the finding of *Walkeria* *uva* in the study area. In contrast, salinity was found to be the only significant factor affecting species richness. The vast majority of bryozoans are marine species with low tolerance to decreased salinity levels [13] and the number of species distributed in transitional zones across salinity gradients is usually low. For example, Ben Ismail et al. [69] reported that species richness of a bryozoan community at marine sites was three times higher than that at lagoon sites in the Mediterranean Basin.

Kuklinski et al. [70] found that water temperature demonstrated no significant relationships with recruitment patterns in bryozoans at King George Island but temperature fluctuations were low (0.76–1.0 °C on average). In our case, seawater temperature range was wider (7.4–13.4 °C) and we found that this factor had a strong negative relationship with bryozoan biomass. This finding is not unexpected because the majority of bryozoans (76%) were Boreo-Arctic and Arctic species less adapted to warm water environments. High summer temperatures are unfavorable for such bryozoan species, resulting in a decrease in their biomasses. This result raises again the importance of climatic factors in driving not only bryozoan but also whole benthic communities in the changing Arctic. The same is relevant for the Southern Ocean, where habitat-forming bryozoan communities are influenced by the combined effects of seasonal ice scour and carbonate chemistry, which, in an increasingly acidified and warming ocean, may put the local bryozoan communities at greater risk [71].

## 5. Conclusions

In this work, bryozoan community structure at two adjoining sites in the intertidal zone of the Barents Sea was linked to local environmental variables, including biogenic element concentrations, silicates, oxygen, wave flow intensity, temperature, and salinity in the period of warming in the Arctic. Biodiversity of bryozoan assemblages and the total biomass were low especially at stations affected by freshwater discharges and exposed to high-temperatures. Redundancy analysis indicated significant contributions of temperature and salinity to biomass and diversity indices with high levels of explained variations: 68% for salinity and 52% for temperature. Other environmental factors were within the range of their multi-annual values and did not affect bryozoans in the study area in summer. Our research revealed a new distribution record for the ctenostome bryozoan *Walkeria* *uva* confirming a global trend to range extension of boreal species in the Arctic. Our data concerning the current status of bryozoan communities in the study area may be considered a reference point for further monitoring whereas the results regarding relationships between diversity and biomass and environmental variables expand our knowledge about the functioning of littoral ecosystems and allow us to predict further changes in the structure of benthic communities associated with ongoing climate change.

## Figures and Tables

**Table 1:** Species composition and biomass of bryozoan species found in the intertidal zone of Yarnyshnaya and Dalnezelenetskaya Bays.

Taxa	Origin	Construction Form	Orientation Form	Biomass, g·m^−2^				Stations
				Min	Max	X	SE	
Cyclostomatida								
*Crisia eburnea* (Linnaeus, 1758)	BA	Ca	Er	0	1.214	0.213	0.141	4,5,6
*Crisiella producta* (Smitt, 1865)	BA	Ca	Er	0	0.72	0.087	0.079	4,5,6
*Filicrisia geniculata* (Milne Edwards, 1838)	B	Ca	Er	0	0.002	0.000	0.000	6
*Oncousoecia diastoporides* (Norman, 1869)	BA	Ca	En	0	0.011	0.001	0.001	6
*Patinella verrucaria* (Linnaeus, 1758)	BA	Ca	En	0	0.002	0.000	0.000	6
Ctenostomatida								
*Alcyonidioides mytili* (Dalyell, 1848)	BA	Fl	En	0	0.002	0.000	0.000	4,6,8
*Amathia gracilis* (Leidy, 1855)	B	Fl	Er	0	0.162	0.018	0.018	6
*Flustrellidra hispida* (O. Fabricius, 1780)	B	Fl	En	0	0.525	0.150	0.073	2,3,4,7
*Walkeria uva* (Linnaeus, 1758)	B	Fl	Er	0	0.001	0.000	0.000	6,8
Cheilostomatida								
*Aquiloniella scabra* (van Beneden, 1848)	BA	Ca	Er	0	0.009	0.001	0.001	6
*Callopora lineata* (Linnaeus, 1767)	BA	Ca	En	0	0.012	0.002	0.001	6,8
*Callopora weslawski* Kuklinski and Taylor, 2006	A	Ca	En	0	0.817	0.134	0.089	3,4,5,6,8
*Celleporella hyalina* (Linnaeus, 1767)	BA	Ca	En	0	0.023	0.004	0.002	3,4,5,6,8
*Cribrilina cryptooecium* Norman, 1903	B	Ca	En	0	1.373	0.198	0.149	3,4,6,8
*Cylindroporella tubulosa* (Norman, 1868)	BA	Ca	En	0	0.011	0.002	0.001	6,8
*Dendrobeania murrayana* (Bean, in Johnston, 1847)	BA	Ca	Er	0	0.004	0.000	0.000	3
*Electra pilosa* (Linnaeus, 1767)	BA	Ca	En	0	0.356	0.103	0.048	3,4,5,6
*Escharella immersa* (Fleming, 1828)	BA	Ca	En	0	0.092	0.010	0.010	6,8
*Eucratea loricata* (Linnaeus, 1758)	BA	Ca	Er	0	4.68	1.009	0.602	4,5,6
*Harmeria scutulata* (Busk, 1855)	A	Ca	En	0	1.676	0.269	0.194	3,4,6,8
*Juxtacribrilina annulata* (O. Fabricius, 1780)	BA	Ca	En	0	0.17	0.024	0.019	6,8
*Microporella arctica* Norman, 1903	B	Ca	En	0	0.14	0.016	0.016	4,6
*Porella alba* Nordgaard, 1906	BA	Ca	En	0	0.003	0.000	0.000	6
*Tegella arctica* (d’Orbigny, 1853)	BA	Ca	En	0	0.08	0.009	0.009	6,8
*Tegella unicornis* (Fleming, 1828)	B	Ca	En	0	0.007	0.001	0.001	8

Note: B—boreal species, BA—Boreo-Arctic species, A—Arctic species, Ca—calcified, Fl—flexible, Er—erect, En—encrusting. *Dendrobeania murrayana* was found on macrophytes, the rest species were found on rocks and boulders.

**Table 2:** Results of SIMPER analysis: contribution of bryozoan species (cut-off 90%) to the total dissimilarity between the groups delineated with cluster analysis.

Species	Average Dissimilarity, %	Contribution, %	Cumulative Contribution, %
*Eucratea loricata*	19.31	21.67	21.67
*Electra pilosa*	10.52	11.81	33.48
*Callopora weslawski*	10.05	11.28	44.77
*Cribrilina cryptooecium*	9.93	11.14	55.91
*Flustrellidra hispida*	8.71	9.77	65.68
*Crisia eburnea*	8.57	9.62	75.30
*Harmeria scutulata*	6.82	7.65	82.94
*Crisiella producta*	3.81	4.28	87.22
*Juxtacribrilina annulata*	2.07	2.33	89.55

**Table 3:** List of environmental variables contributed to the RDA models based on the bryozoan diversity and biomass data in the coastal Barents Sea.

Diversity				Biomass			
Variable	LambdaA	F	*p*	Variable	LambdaA	F	*p*
S	68	14.77	0.002	T	52	7.52	0.001
P-PO_4_	9	2.26	0.140	O_2_	8	1.15	0.341
Si-SiO_3_	5	1.35	0.276	WFII	7	1.05	0.385
T	5	1.84	0.235	N-NO_2_	7	1.15	0.354
N-NO_3_	4	1.23	0.33	N-NO_3_	11	2.27	0.163
N-NO_2_	3	1.19	0.394	S	6	1.24	0.376
WFII	2	0.53	0.586	Si-SiO_3_	3	0.47	0.687
O_2_	4	0	1	P-PO_4_	6	1	1

Note: T—temperature (°C), S—salinity, O_2_—oxygen concentration (mg L^−1^), SiO_3_—silicates (µg·L^−1^), P-PO_4_—phosphates (µg·L^−1^), N-NO_2_—nitrites (µg·L^−1^), N-NO_3_—nitrates (µg·L^−1^), and WFII—wave flow intensity index, LambdaA—explained variation, %, F—pseudo F-ratio, p—probability level.

## Data Availability

The data are available on request from the corresponding author.
